# Anti-mycobacterial activity correlates with altered DNA methylation pattern in immune cells from BCG-vaccinated subjects

**DOI:** 10.1038/s41598-017-12110-2

**Published:** 2017-09-26

**Authors:** Deepti Verma, Venkata Ramanarao Parasa, Johanna Raffetseder, Mihaela Martis, Ratnesh B. Mehta, Mihai Netea, Maria Lerm

**Affiliations:** 1 Division of Microbiology and Molecular Medicine, Linköping University, SE-58185 Linköping, Sweden; 2 NBIS (National Bioinformatics Infrastructure Sweden), ScilifeLab, Division of Cell Biology, Department of Clinical and Experimental Medicine, Faculty of Medicine and Health Sciences, Linköping University, SE-581 85 Linköping, Sweden; 3 Clinical Immunology, Department of Clinical and Experimental Medicine, Faculty of Medicine and Health Sciences, Linköping University, SE-58185 Linköping, Sweden; 4 Department of Internal Medicine, Radboud University Medical Center, Geert Grooteplein 8, Nijmegen, The Netherlands

## Abstract

The reason for the largely variable protective effect against TB of the vaccine Bacille Calmette-Guerin (BCG) is not understood. In this study, we investigated whether epigenetic mechanisms are involved in the response of immune cells to the BCG vaccine. We isolated peripheral blood mononuclear cells (PBMCs) from BCG-vaccinated subjects and performed global DNA methylation analysis in combination with functional assays representative of innate immunity against *Mycobacterium tuberculosis* infection. Enhanced containment of replication was observed in monocyte-derived macrophages from a sub-group of BCG-vaccinated individuals (identified as ‘responders’). A stable and robust differential DNA methylation pattern in response to BCG could be observed in PBMCs isolated from the responders but not from the non-responders. Gene ontology analysis revealed that promoters with altered DNA methylation pattern were strongly enriched among genes belonging to immune pathways in responders, however no enrichments could be observed in the non-responders. Our findings suggest that BCG-induced epigenetic reprogramming of immune cell function can enhance anti-mycobacterial immunity in macrophages. Understanding why BCG induces this response in responders but not in non-responders could provide clues to improvement of TB vaccine efficacy.

## Introduction

Tuberculosis (TB) is one of the leading causes of death from infectious disease worldwide. The only available TB vaccine is Bacille Calmette Guérin (BCG), which has low efficacy against adult pulmonary TB. Since the pulmonary manifestation of TB is the major source of transmission, an effective prevention strategy against pulmonary TB is urgently needed. New vaccine candidates are based on the addition of *Mycobacterium tuberculosis* (Mtb) antigens to the BCG regimen, however none has yet provided better protection than BCG^1^.

Effective vaccine development is facilitated by the use of immunological correlates, which may predict the outcome of vaccination. For TB vaccine research, the general strategy is to measure mycobacteria-specific T cell responses, however, earlier studies have failed to prove such a correlation^2^, suggesting that other protective mechanisms may be at play.

Evidence is accumulating that during lifetime, the human immune system is gradually epigenetically reprogrammed. Both DNA methylation changes (e.g. demethylation of transcription start sites enhances transcriptional activity^3^) and histone modifications (e.g. histone acetylation/methylation increases or decreases the accessibility of DNA to transcription factors^4^) contribute to this phenomenon. Post-translational histone modifications in immune cells have been shown to result in an enhanced response to microbial stimuli^5,6^. Furthermore, altered DNA methylation has been demonstrated in a subset of natural killer (NK) cells isolated from cytomegalovirus-infected humans^7^. However, nothing is known about genome-wide DNA methylation changes in response to vaccination.

Here, we investigated the effects of BCG vaccination *in vivo* on the DNA methylome of human immune cells. We demonstrate that the pattern of DNA methylation is altered in cells isolated from a subset of individuals classified as ‘responders’ based on their enhanced macrophage capacity to restrict growth of Mtb. A substantial part of the responders’ gene promoters that showed the strongest alteration in DNA methylation were found to be part of immune-related pathways. The findings suggest that altered DNA methylation could be taken into consideration in studies investigating why individuals respond differently to the BCG vaccine^8,9^.

## Results

### Enhanced anti-mycobacterial activity in MDMs from a subset of BCG-vaccinated individuals

**Figure 1:**
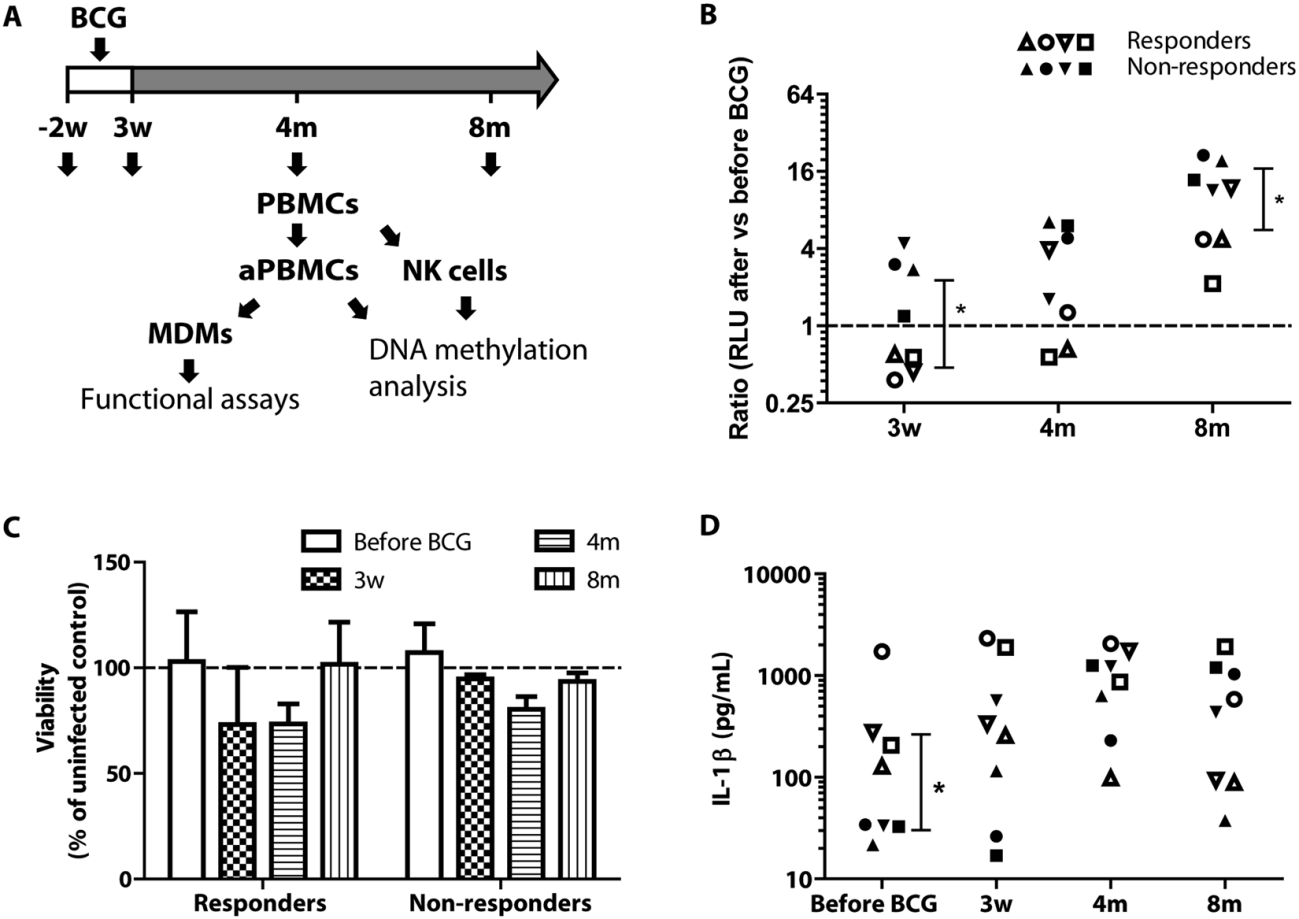
Anti-mycobacterial activity and cytokine response to mycobacterial infection of MDMs. (**A**) Diagram of the blood collection timeline. Blood was collected 1–2 weeks before (−2W) and 3 weeks, 4 months and 8 months post-BCG vaccination. PBMCs were isolated for DNA isolation and methylation analysis or used to prepare monocyte-derived macrophages for infection with luciferase-expressing Mtb. (**B**) MDMs’ anti-mycobacterial capacity (as determined by the ratio of bacterial numbers at D4/D0 in each individual experiment) after vs before BCG vaccination were determined using luminometry. (**C**) MDM cell viability at 4 days of infection with H37Rv compared to uninfected cells from the same day as determined by calcein-AM fluorescence. (**D**) For cytokine analysis, the medium supernatant was collected at day 4 of infection and analyzed by cytometric bead array. Empty symbols represent responders while the solid symbols represent the non-responders. Statistical significance compared to before BCG was determined using Student’s one-sample t test, comparison between responders and non-responders was done using two-tailed Student’s t test and cytokines were compared using Mann-Whitney U test. *p < 0.05, **p < 0.01,***p < 0.001.

To compare the anti-mycobacterial activity of monocyte-derived macrophages (MDMs) isolated one week before and 3 weeks, 4 months and 8 months after BCG vaccination, we collected peripheral blood from 8 BCG-vaccinated individuals (Fig. 1A) at the given time points. MDMs differentiated from adherent peripheral blood mononuclear cells (aPBMCs) were infected with virulent Mtb. For each time point of blood sampling, the cells were lysed at day 0 and day 2 after infection and the relative Mtb growth (day 2/day 0) was determined by luminometry. Analysis of the ability of Mtb to replicate in the cells relative to the pre-BCG time point was compared for each donor (each donor was his/her own control, applying a case series approach permitting lower power^10^). The results showed that MDMs isolated 3 weeks post-BCG from a subset of individuals (n = 4) displayed enhanced anti-mycobacterial capacity as compared to their MDMs isolated before vaccination (p = 0.002, one-sample t test) (Fig. 1B & S1). Based on this observation, and using a strategy that has been applied in previous studies^9,11^, we divided the subjects into two groups classified as responders (n = 4, enhanced anti-mycobacterial activity) and non-responders (n = 4). These two groups significantly differed from each other at 3 weeks post-BCG (p = 0.03, Student’s two-tailed t test with Bonferroni correction) (Fig. 1B) and also displayed a significant difference over time (p = 0.001, ANOVA). The ability to control Mtb gradually waned, however, after 8 months, the responders were still superior to non-responders in mycobacterial control (p = 0.03, Student’s two-tailed t test with Bonferroni correction) (Fig. 1B). As previously reported, Mtb infection of MDMs caused cell death^12^, however, the viability was similar in all conditions and time points (p = 0.09, Student’s two-tailed t test with Bonferroni correction Fig. 1C).

We next analyzed a set of macrophage-associated cytokines in the supernatants from the Mtb-infected MDMs and observed that the levels of interleukin (IL)-1β were significantly higher in the responders (IL-1β responders = 584.2 pg/ml, IL-1β non-responders = 30.39 pg/ml) at the time point before BCG vaccination (p = 0.02, Mann Whitney U test, corrected). All individuals displayed an increased IL-1β response after BCG vaccination, however the time point for the maximal response varied (Fig. 1D). The other analyzed pro-inflammatory cytokines (IL-6, IL-10, IL-12, tumor necrosis factor (TNF)-α and interferon (IFN)-γ were not significantly different between the groups (not shown). In all unstimulated samples, the cytokines were below detection limit (not shown).

### Genome-wide DNA methylation profiling reveals altered DNA methylation in responders

We next turned to investigate whether BCG would alter the DNA methylome of the PBMCs of vaccinated individuals. To this end, we analyzed the methylation pattern of DNA isolated from the subjects’ aPBMCs. The global DNA methylation of CpG sites passing the quality filtering was plotted according to the genomic locations. The overall DNA methylation quantified by the β value was found to be lower in the promoter region as compared to the gene body, 3’UTR and the intergenic regions (regions unannotated by Illumina, not shown).

**Figure 2:**
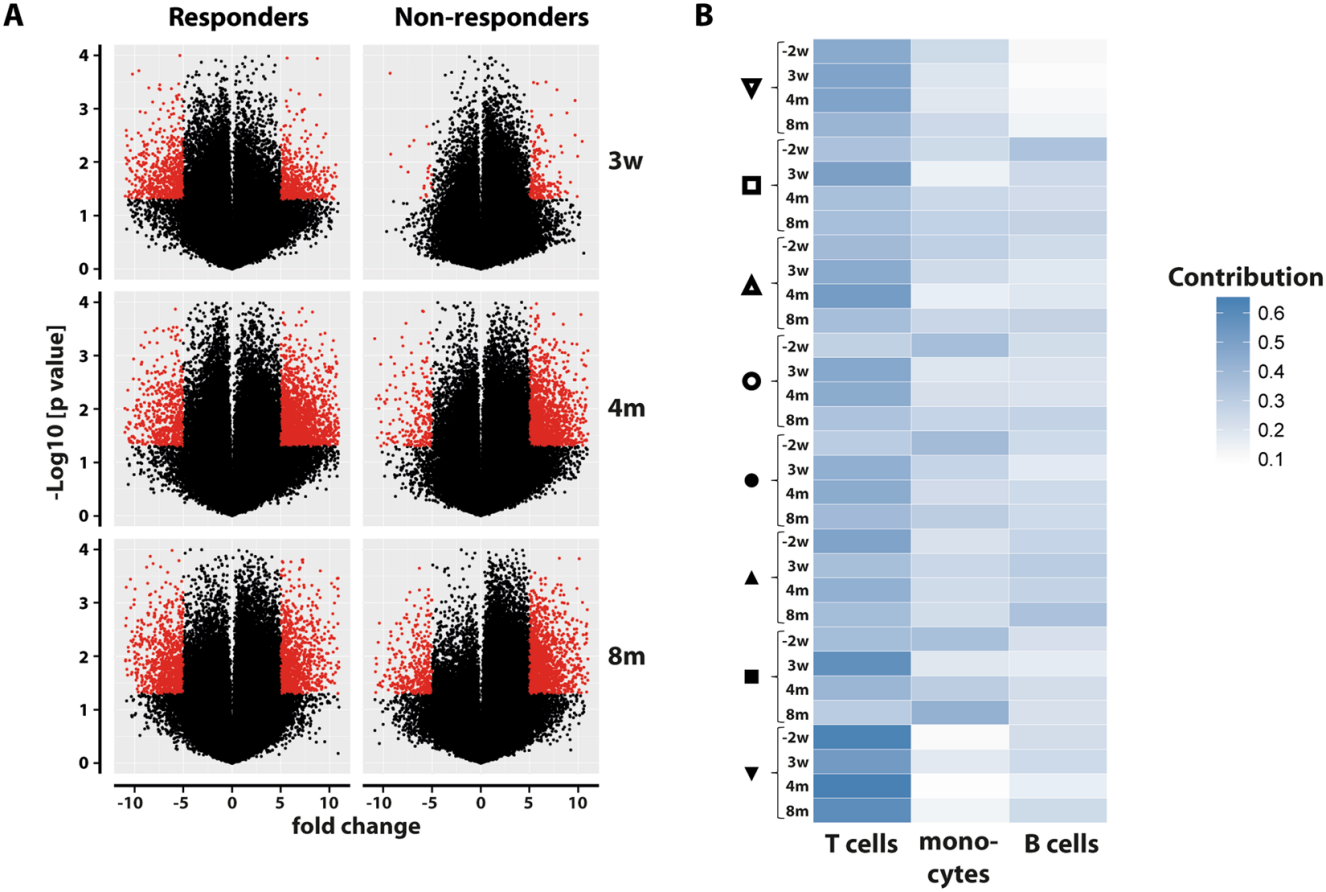
Global DNA methylation changes in aPBMCs of responders and non-responders. (**A**) Volcano plot showing the differential DNA methylation at 3 weeks, 4 months and 8 months compared to the time point before BCG. The red dots represent the promoters where the difference in methylation (β values) as compared to before BCG was greater than 5-fold. Red dots on the left represent the promoters with a loss of methylation while those on the right represent the promoters with a gain of methylation. (**B**) Heat map showing the individual cell types’ contribution to DNA methylation. Darker color represents a higher contribution.

At 3 weeks post-BCG, a distinct alteration of methylation (compared to the time point before BCG) was observed in the DNA isolated from the responders’ aPBMCs but not to the same extent in the non-responders’ DNA (Fig. 2A). 540 promoters displayed a more than 5-fold loss of methylation (predicted activation) in the responders (p < 0.01, Student’s paired t test), while only 20 promoters losing methylation were observed in the non-responders group at this early time point. Using the 5-fold cut-off, 477 and 183 promoters gaining methylation (predicted inactivation) were observed in responders and non-responders, respectively. Applying the same high stringency to the data obtained at the later time points (before BCG vs 4 months and 8 months, respectively) revealed a persistent differential DNA methylation in the responders. At 4 and 8 months, a substantial gain of DNA methylation was observed in the non-responders and a gradually increasing loss of methylation as compared to the time point before BCG was also detected (Fig. 2A). Gender breakdown did not show any pattern (two males and two females among the responders, not shown).

The aPBMCs were analyzed by flow cytometry and shown to be composed of CD3^+^ T cells (~40–50%), CD14^+^ monocytes (~40%) and CD19^+^ B cells (~10%, not shown). In order to accurately predict the contribution of these individual cell types to the overall β values, we made use of the recently described Houseman algorithm^13^. The T cells contributed the most followed by the monocytes and B cells. The division of the datasets into responders and non-responders did not reveal any major difference between the contributing cell types at the different time points (Fig. 2B).

**Figure 3:**
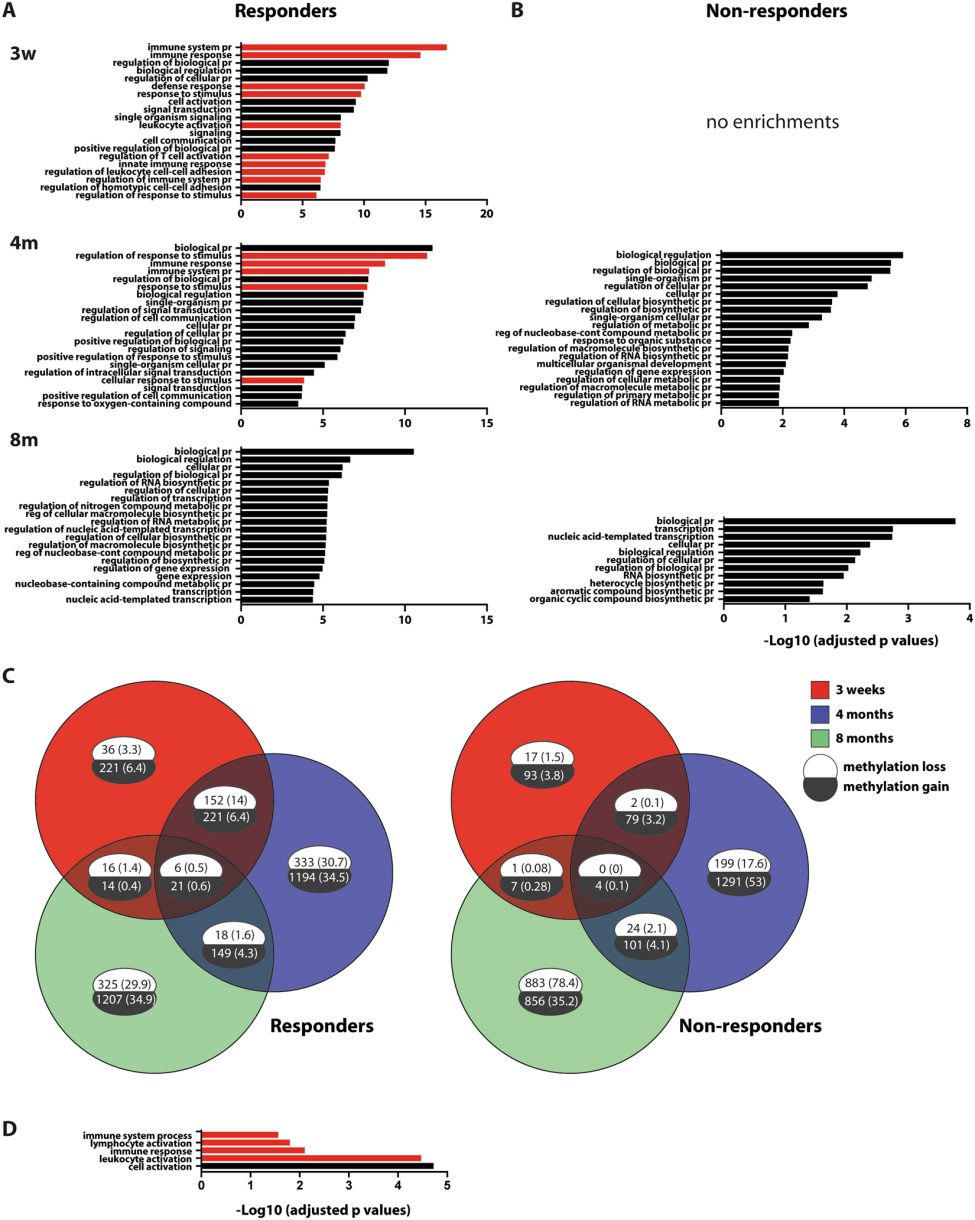
Gene Ontology analysis of differentially methylated promoters in responders and non-responders. (**A**–**B**) Gene ontology analysis using PANTHER with the DNA methylation data at 3 weeks, 4 months and 8 months describing the overrepresented biological pathways on the Y- axis and log 10 adjusted p values (<0.01) on the X- axis. Immune function-related pathways are represented as red bars. No enrichments were found for the non-responders at 3 weeks’ time point. (**C**) Venn diagram showing the overlap of differentially methylated promoters (represented as absolute numbers) at 3 weeks (pink), 4 months (blue) and 8 months (green). Numbers within brackets show the corresponding percentages of total hypo or hyper-methylated promoters. (**D**) gene ontology analysis of the promoters overlapping between the 3 weeks’ and 4 months’ time point in the responders. No corresponding enrichments were found for the non-responders. Promoters with a loss of methylation are shown in the white bubbles and those with a gain of methylation are shown in the black bubbles. Abbreviations: pr = process, reg = regulation.

To interpret the functionality of the observed changes, the differentially methylated promoters were annotated using the PANTHER database^14^. The annotation revealed a significant overrepresentation of GO terms related to immune system and defense response in the responders at 3 weeks post-BCG. Figure 3A visualizes the enriched GO terms in the responders’ data set at 3 weeks, these include terms like T cell activation, innate immune response, leukocyte activation and adhesion. No overrepresentations were found for the non-responder group at this time point. Immune process-related pathways continued to be overrepresented at 4 months in the responders and enrichments were found for cell communication, response to stimulus and signal transduction (Fig. 3A). At this time point, the non-responders displayed overrepresentations for the GO terms metabolic process and signal transduction (Fig. 3B). No immune-related overrepresentation could be found in either the responders or the non-responders at 8 months post-BCG.

To determine whether an altered DNA methylation pattern of individual promoters persisted throughout the 8 months post BCG vaccination, the data obtained from the three time points after BCG vaccination was combined in a Venn diagram (Fig. 3C). The analysis revealed that a set of 28 promoters, 6 with a loss of methylation and 21 with a gain, persisted in the responders throughout 8 months (Fig. 3C and Tables 1 and 2). Among the promoters with persistently reduced methylation were adenylate cyclase 3 (*ADCY3)* and IFN-γ *(IFNG)*. None of the promoters in the non-responders displayed reduced methylation at all time points (Fig. 3C). Using PANTHER, we also determined the pathways to which the promoters belonged that were overlapping between the 3 weeks and 4 months’ time points of the responders (152 with reduced and 221 with increased methylation, respectively) (Fig. 3D). The analysis revealed dominance of immune-related pathways.

**Table 1:** Promoters displaying persistent differential methylation at all time points: Responders.

GENE SYMBOLS	DESCRIPTION
IFNG	Interferon gamma
RASAL1	RasGAP-activating-like protein 1
GIMAP7	GTPase IMAP family member 7
ADCY3	Adenylate cyclase type 3
ATXN1	Alterernative reading frame 1
DIABLO	Direct IAP-Binding Protein With Low PI

Loss of DNA methylation.

**Table 2:** Promoters displaying persistent differential methylation at all time points: Responders: Gain of DNA methylation.

GENE SYMBOLS	DESCRIPTION
TLR6	Toll-like receptor 6
SRD5A2	3-oxo-5-alpha-steroid 4-dehydrogenase 2
SOX5	Transcription factor SOX-5
GNG7	G nucleotide-binding protein G(I)/G(S)/G(O) subunit gamma-7
SBNO2	Protein strawberry notch homolog 2
SULT1C	Sulfotransferase 1 C
NFKBIE	NF-kappa-B inhibitor epsilon
TRIM2	Tripartite motif-containing protein 2
GPR84	G-protein coupled receptor 84
SPATS2	Spermatogenesis-associated serine-rich protein 2
CD59	Complement regulatory protein
ATXN1	Alternative reading frame 1
NCOR2	Nuclear receptor co-repressor 2
ADARB1	Adenosine deaminase
LOC404266	Undefined function
SRGAP3	SLIT-ROBO RHO GTPase-activating protein 3
PIWIL2	PIWI-like 2
SPG20	Spastic paraplegia 20
TSPAN4	Tetraspanin4
CSGALNACT1	Chondroitin sulphate N-acetylgalactosaminyltransferase 1

### Reduced methylation of promoters in responders’ NK cells 3 weeks post-BCG

**Figure 4:**
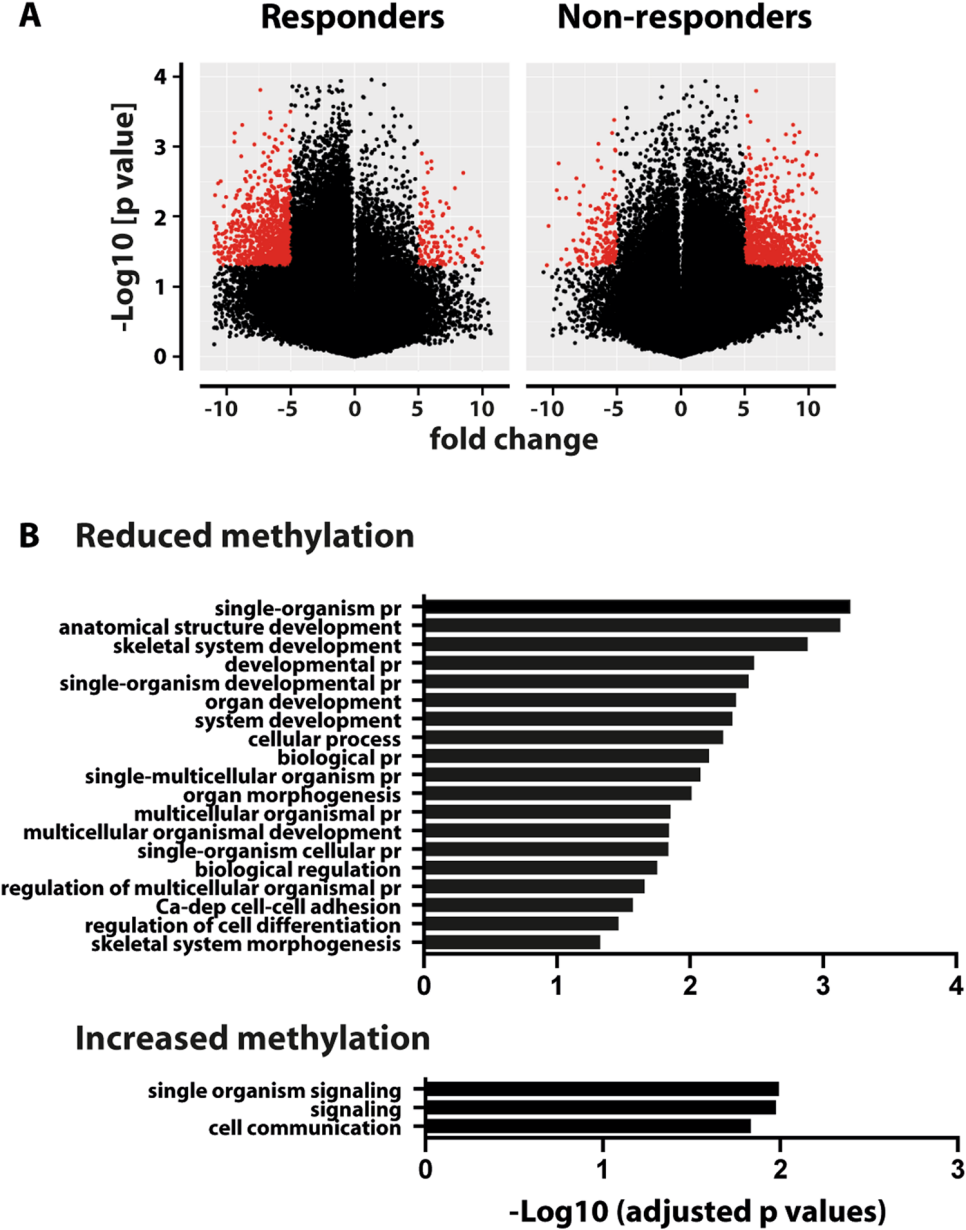
Global DNA methylation changes in NK cells from responders and non- responders (**A**) Volcano plot showing the DNA methylation pattern in NK cells at 3 weeks compared to before BCG. The red dots represent the promoters where the difference in methylation β values as compared to before BCG was greater than 5-fold. Red dots on left represent the promoters with a loss of methylation while those on right represent the promoters with a gain of methylation. (**B**) Gene ontology analysis of differentially methylated promoters in NK cells at three weeks. The graphs depict the overrepresented biological pathways on the Y- axis and log 10 adjusted p values (<0.01) on the X- axis.

NK cells, which are innate lymphocytes, have been shown to have memory characteristics^15^. In order to determine whether BCG vaccination also affected DNA methylation in NK cells, DNA was prepared from the subjects’ NK cells isolated before BCG and 3 weeks post-BCG vaccination and analyzed using the Illumina platform. Distinctive DNA methylation profiles for responders and non-responders were observed with a predominant methylation loss in the responders and a gain of methylation in the non-responders at 3 weeks post-BCG (Fig. 4A). The gene ontology analysis revealed that the gain in DNA methylation in the non-responders is overrepresented for cell communication and cell signaling (Fig. 4B). No overrepresentation of immune-related terms could be identified in the responders’ promoters, however, the promoters of *NLRP3* and *caspase-1*, both key components of the IL-1β producing inflammasome, displayed reduced methylation. Further, the responders showed reduced methylation of promoters of signaling proteins like protein tyrosine phosphatases, ATP transporters, cytolytic molecules (granzymes) and inflammation mediators. Among the non-responders, a gain of methylation was observed in promoters regulating NK cell function such as *NCR1* (Natural cytotoxity receptor 1), *IFNG*, type III IFN, granzyme K as well as in the ATPases and ATP binding cassettes, interleukins and signaling molecules.

## Discussion

The aim of the present study was to investigate whether BCG vaccination affects the DNA methylome of immune cells and whether this could be linked to an enhanced anti-mycobacterial activity in Mtb-infected macrophages. We took an omics approach to study global DNA methylation changes in the immune cells of BCG-vaccinated individuals and combined this with functional assays reflecting innate immunity directed towards Mtb. We show that immune cells from the individuals, whose macrophages showed increased capacity to control Mtb after BCG vaccination, displayed differential methylation in a large number of promoters at 3 weeks after vaccination. Thus, as described in previous work^9^, BCG induced a dichotomous response in our small, well-defined study group.

Since it has proven challenging to extrapolate the functional consequences of genome-wide DNA methylation alterations, we focused on altered DNA methylation in the proximity of translational start sites (TSS) regions, 5’UTR and the first exon, as changes associated with these regions have been shown to be reflected in gene expression^16,17^. The gene ontology analysis revealed that immunity-related pathways were enriched for differentially methylated promoter-proximate regions at 3 weeks after BCG in the responders’ immune cells. This effect persisted through 4 months in accordance with a recent study showing BCG-induced histone modifications in monocytes 3 months post-vaccination^18^. In addition, the 152 promoters with reduced and 221 with enhanced DNA methylation, respectively, that were stable from 3 weeks through 4 months post-vaccination were strongly enriched for immune-related pathways. We detected persistently reduced methylation of the promoter of the adenylate cyclase (AC) gene *ADCY3* at all time points in the responders. Of note, recent studies have shown that *ADCY3* is linked to the increased cytokine production that accompanies trained immunity^19^. In addition, the promoter of *IFNG* remained in a reduced methylated state in the responders throughout the duration of the study. IFN-γ is well-established as one of the most crucial factors that allow the immune system to control mycobacterial infection^6,20^. It could be argued that these persisting effects of BCG in the responders are due to the presence of BCG organisms in the body, thus being a more direct effect of BCG on the immune cells rather than an epigenetic memory of the exposure. Indeed, the persistence of BCG in the body has been shown to last for about one month post-BCG^21^, hence, the present study cannot rule out that this contributes to the observed effects.

We demonstrate that individuals, whose MDMs had an inherent ability to respond to Mtb exposure by an increased IL-1β production responded to the vaccine by enhancing MDM efficacy in controlling the replication of Mtb. This is reminiscent of the increased antifungal killing in models of trained immunity induced in macrophages by β-glucan^22^. The present study does not unravel whether IL-1β acts upstream or downstream of the induction of epigenetic reprogramming. However, the involvement of this cytokine for the establishment of antimicrobial defense mechanisms via induction of trained immunity is supported by studies showing that injection of IL-1β before infection can protect against infection with *Pseudomonas aeruginosa* or *Candida albicans* ^23,24^.

While the adaptive immune responses to BCG are very well-studied, the understanding of the effects of this vaccine on other aspects of immunity is only emerging. Early studies conducted in the years after the introduction of BCG in vaccination programs showed that BCG protected against TB-unrelated child mortality^25^ and more recent studies have confirmed these findings^26–29^. These effects have been suggested to be induced through epigenetic reprogramming^30,31^. Trained immunity is generated independently of functional T- and B cells and involves histone modifications induced via a Nod2-mediated pathway^5^. The present study adds DNA methylation alterations to this concept.

NK cells were recently found to respond to BCG vaccination by an increase in IL-1β production^15^. Here, responders’ NK cells displayed decreased methylation of both *NLRP3* and *caspase-1* promoters, which is indicative of facilitation of IL-1β production. NK cells are suggested to have a role in the early clearance of Mtb infection due to their early appearance and evidence in Mtb killing and macrophage apoptosis^32^.

The small sample size of the present study limits extrapolation of the findings, however, for any studies on changes in the DNA methylome, intervention studies like this allows the comparison of data from each individual before vs. after intervention, which allows interpretation of data from a smaller sample size. Since DNA methylome patterns varies greatly by ethnicity, age, environmental factors and possibly seasonal changes, it is attractive to focus on a well-defined study group assessed at several time points before and after an intervention like vaccination. From the present study, no conclusions can be drawn on a possible correlation between BCG-induced epigenetic changes and vaccine efficacy. Hence, future studies in TB-endemic settings are warranted to assess whether altered epigenetic changes in response to BCG vaccination could be predictive of protection against TB.

## Materials and Methods

### Ethics statement

The study protocol was approved by the local ethics committee in Linköping (Etikprövningsnämnden #2013/203-31). All individuals signed an informed consent to participate in the study. All experimentation was performed in accordance with the Declaration of Helsinki. All samples were deidentified making any tracing of subjects impossible.

### Study subjects

70 ml blood were collected in heparinized tubes 1–2 weeks prior to BCG vaccination and 3 weeks, 4 months and 8 months after the vaccination from 8 healthy individuals. The subjects were all non-smoking, PPD-negative, Swedish-born Caucasians (20–35 years, mean age 24 years, 5 males) with normal BMI and no history of BCG vaccination.

### Bacteria

Mtb H37Rv (ATCC) carrying the luciferase-encoding pSMT1 plasmid was cultured as previously described^33^.

### Isolation of mononuclear cells

PBMC were separated by density centrifugation on Lymphoprep as previously described^34^ and either frozen (at −150 ^°^C, 3 months and 8 months’ time point) or seeded (the ‘before BCG’ and the ‘3 weeks post BCG’ time points) in cell culture flasks, allowing enrichment of adhesive cells. Following adherence, the non-adherent lymphocytes were washed off. Cells were either trypsinized to isolate genomic DNA or allowed to differentiate into macrophages as previously described^34^. Phenotyping of the adherent cells was performed by flow cytometry (not shown).

### Purification of NK cells

NK cells were purified from the non-adherent fraction of the PBMCs by magnetic bead separation using the human NK cell isolation kit (Miltenyi Biotec GmbH, Gladbach, Germany). Purity as determined by flow cytometry was >95%.

### Flow cytometry

Phenotyping of the adherent cells was performed by flow cytometry. Cells were labelled using mouse anti-human monoclonal antibodies (CD3-FITC, CD56-PE, CD14-APC, CD16b-PE and CD19-PE, BD Biosciences, San Jose, CA, USA) for 30 mins at 4 °C before washing and resuspension in PBS and detected using a Gallios flow cytometer (Beckman Coulter, USA). Analysis of the flow cytometric data was performed with Kaluza Analysis software version 1.3 (Beckman Coulter, USA).

### Mycobacterial replication and macrophage viability

Infection using luciferase-expressing H37Rv at a multiplicity of infection of 5 was performed as previously described^35^ and bacterial load and cell viability was determined at day 0 and day 4. Cell supernatants were collected, centrifuged and stored at −70 °C for cytokine determination.

### Cytokine measurements

The cytokines were determined using the human flex sets for Cytometric Bead Array (IFN-γ using Human IFN-γ Enhanced Sensitivity Flex Set, Becton Dickinson), followed by 30 min 4% paraformaldehyde fixation. Data were acquired on a Gallios Flow Cytometer and analyzed using its Kaluza software (Beckman Coulter).

### DNA extraction and Infinium Methylation Assay

DNA was isolated from NK cells and the aPBMCs using QiAmp DNA isolation kit following the instructions. DNA concentration was measured by Qubit fluorometer. DNA samples were bisulphite converted, amplified, fragmented and hybridized to an Illumina Infinium Human Methylation 450 K Bead Chip and scanned.

### Bioinformatic analysis

The raw data was imported into R version 3.2.2 and analyzed using the Bioconductor Minfi package. Background correction and within-array subset quantile normalization were implemented. Probes overlapping with SNPs, or having a detection p value > 0.01 as well as the probes present on the X and Y chromosomes were excluded. The β values (0–1) were derived from the processed data defined as the ratio of methylated probe intensity to the sum of methylated and unmethylated probe intensity. The methylation for genomic regions was calculated as average β value for all probes located within the regions annotated by Illumina: Transcription start site (TSS)200, TSS1500, 5’untranslated region (UTR), 1^st^ exon, gene body, 3’UTR and intergenic (unannotated) region. Promoters were defined as covering the TSS, 5’UTR and first exon. Differentially methylated (DM) loci were determined using a paired Student’s t-test and were selected if the fold change in β value exceeded 5% with corresponding p values less than 0.01. The differential DNA methylation analysis has been performed with support from NBIS (National Bioinformatics Infrastructure Sweden).

### Gene function annotation and pathways analyses

The functions of differentially methylated promoters were annotated using the PANTHER database (http://www.pantherdb.org/). The official gene symbols were used as input to calculate the statistical over-representation of biological process GO terms using a binomial test. Only pathways with a Bonferroni corrected p value < 0.01 were considered significant and top 20 pathways of them are presented.

### Estimating contribution of different cell types

To estimate the relative proportion of different cells types in the aPBMC samples the Houseman correction was implemented in Minfi (FlowSorted.Blood.450 k), which is especially developed for Illumina 450 K arrays^13,36^. The algorithm combines the user’s input intensity data with the flow-sorted data, normalizes the data and then estimates the cell composition^37^.

### Data availability statement

The Illumina datasets will be available at the Gene Expression Omnibus database at NCBI (https://www.ncbi.nlm.nih.gov/geo/).

## Electronic supplementary material

Supplemetary figure S1
