# Supplementary material for: Anti-mycobacterial activity correlates with altered DNA methylation pattern in immune cells from BCG-vaccinated subjects
Source: Sci Rep. 2017 Sep 26;7:12305. doi: 10.1038/s41598-017-12110-2 (PMC5615063; doi:10.1038/s41598-017-12110-2)
Supplement: Supplementary file 1 — Supplemetary figure S1 [file 41598_2017_12110_MOESM1_ESM.doc]

**Supplementary information**

**SREP-17-20208A**

Anti-mycobacterial activity correlates with altered DNA methylation pattern in immune cells from BCG-vaccinated subjects

By Deepti Verma, Venkata Ramanarao Parasa, Johanna Raffetseder, Mihaela Martis, Ratnesh B. Mehta, Mihai Netea and Maria Lerm

**
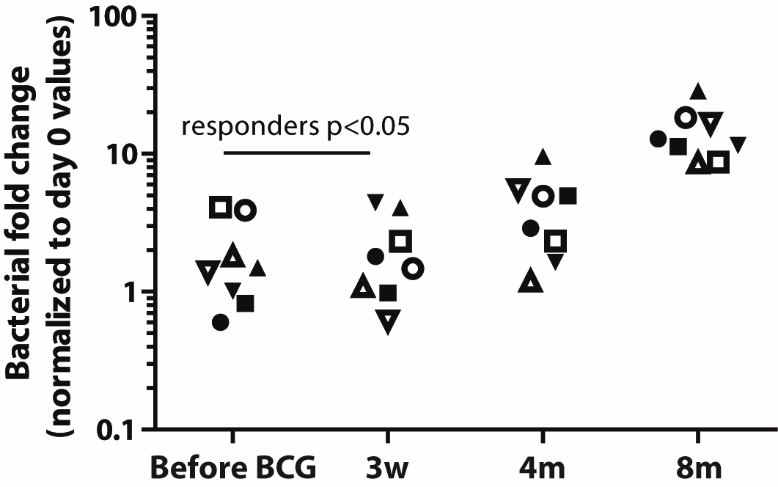
**

**Figure S1.** MDMs’ capacity of mycobacterial control shown as ratio of bacterial numbers normalized to day 0 values were determined using luminometry. Arbitrary luminescence units (ALU) for cell lysate and cell culture medium supernatant were added to give totals. Empty symbols represent responders while the solid symbols represent the non-responders.
